# Papillary Muscle Rupture During Percutaneous Transvenous Mitral Commissurotomy: An Uncommon Scenario Exempt From Emergency Surgical Conversion

**DOI:** 10.1155/cric/5005780

**Published:** 2025-08-21

**Authors:** Hiroki Okamoto, Atsushi Hayashi, Kohei Asada, Misato Kodama, Yousuke Higo, Noriaki Yagi, Yoshihisa Nakagawa

**Affiliations:** Department of Internal Medicine, Division of Cardiovascular Medicine, Shiga University of Medical Science, Otsu, Shiga, Japan

## Abstract

Papillary muscle rupture is a rare but serious complication during percutaneous transvenous mitral commissurotomy (PTMC). In many cases, it leads to acute significant mitral regurgitation (MR), requiring urgent surgical repair. We performed PTMC for a 49-year-old woman with symptomatic moderate rheumatic mitral stenosis. Initial balloon inflation resulted in papillary muscle rupture; however, it did not induce leaflet prolapse and worsening of MR. Finally, we succeeded in achieving a mean pressure gradient of less than 5 mmHg without worsening of MR through several balloon inflations. We experienced a rare case where papillary muscle rupture occurred during PTMC, but there was no development of acute significant MR, and salvage surgery was not required.

## 1. Introduction

Percutaneous transvenous mitral commissurotomy (PTMC) is a less invasive treatment for symptomatic and significant rheumatic mitral stenosis (MS) [1]. Generally, for this procedure, an Inoue balloon is employed via a transseptal approach by splitting open fused commissures to relieve stenosis, with an excellent outcome. Although papillary muscle rupture is a rare complication of PTMC, once it occurs, salvage mitral valve surgery is necessary to manage acute mitral regurgitation (MR) [2–4]. Here, we report a rare case of papillary muscle rupture that occurred during PTMC, demonstrating an unconventional clinical course.

## 2. Case Presentation

**Figure 1:**
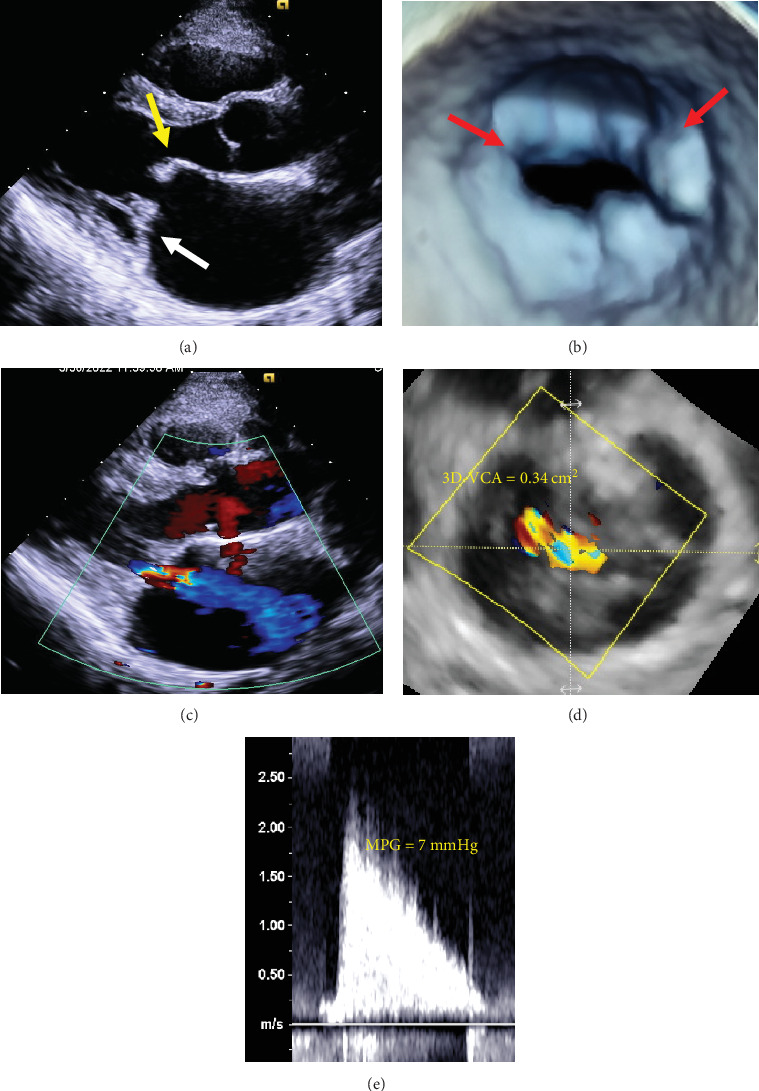
Two-dimensional (2D) TTE, parasternal long-axis view (a), and three-dimensional (3D) TEE, en face view from the left atrium (b), demonstrate the “hockey stick” appearance of the anterior leaflet (*yellow arrow*), poor mobility of the posterior leaflet (*white arrow*), and fusion of the commissures (*red arrows*). Continuous-wave Doppler across a stenotic mitral valve by TTE (c). Mean pressure gradient of 7 mmHg indicates moderate MS. 2D TTE color Doppler (d) and 3D TEE multiplanar reconstruction imaging (e) at mid-systole demonstrate central moderate MR. MPG, mean pressure gradient; 3D-VCA, three-dimensional vena contracta area.

A 49-year-old woman with persistent atrial fibrillation (AF) and atrial tachycardia (AT) was referred to our institution for catheter ablation. Transthoracic echocardiography (TTE) showed typical features of rheumatic MS, with “hockey stick” appearance of the anterior mitral leaflet and immobility of the posterior mitral leaflet in the long-axis view and bicommissural fusion in the short-axis view (Figure 1a,b). Doppler echocardiography demonstrated moderate MR (three-dimensional vena contracta area = 0.34 cm^2^; Figure 1c,d) and moderate MS (mean pressure gradient = 7 mmHg at a heart rate of 60–70 beats/min with AF; mitral valve area measured by continuity equation = 1.1 cm^2^; Figure 1e). Warfarin and enalapril were administered. The patient initially underwent cardioversion for AF, but AF recurred within a few days. Consequently, we decided to perform invasive treatment of the mitral valve because she had dyspnea on exertion (New York Heart Association Functional Class II). The echocardiographic features of the mitral valve included immobility of the posterior mitral leaflet tip and mid-portions with the entire leaflet thickened, but no remarkable calcification of the leaflets and thickening of the subvalvular apparatus, giving a Wilkins score of 8. Therefore, the mitral valve apparatus was suitable for PTMC, although she had moderate MR, which could be a contraindication for PTMC. The MR jet appeared to be central, originating from the center of the mitral leaflets due to mitral annular dilatation rather than leaflet degeneration (Figure 1b). We evaluated the patient's mitral valve morphology preoperatively using three-dimensional TEE. The measurements indicated an anterior-to-posterior diameter of the mitral annulus of 32.4 mm and commissure-to-commissure diameter of 32.0 mm. This finding suggested that MS limited annulus dilatation only in the anterolateral–posteromedial direction, not in the posterior direction. In addition, the length of the mitral anterior and posterior leaflets was measured at 19.3 and 13.3 mm, respectively. These measurements showed relatively short leaflet length or limited leaflet elongation, which was consistent with rheumatic degeneration. These findings—prominent posterior–directed mitral annular dilation and short leaflet lengths associated with rheumatic degeneration—suggested that the cause of MR was insufficient leaflet coaptation due to an imbalance between the mitral annulus and the leaflets. Accordingly, we determined that PTMC was a valid option along with mitral valve replacement (MVR) because we anticipated a decrease in MR following the procedure due to the reduction in annular area. After presenting the option of MVR, we explained to the patient that PTMC also had the potential to improve both MS and MR. She subsequently decided to undergo PTMC. An Inoue balloon with a maximum inflated diameter of 28 mm (Toray, Tokyo, Japan) was selected according to the patient's height of 161 cm. Our preprocedural strategy was as follows: inflation was started at 24-mm diameter, increased incrementally by 1 mm with each inflation, and stopped when the mean pressure gradient fell below 5 mmHg.

**Figure 2:**
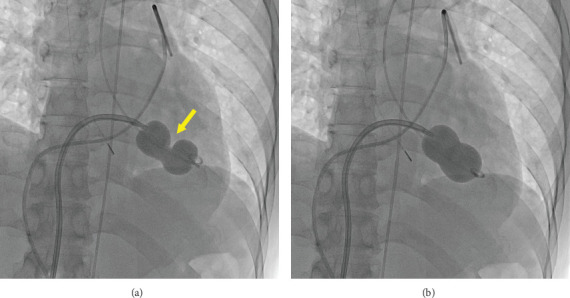
X-ray fluoroscopy during quick inflation of the Inoue balloon demonstrating a deep indentation of the balloon (a, *arrow*) shortly followed by full extension of the balloon (b).

**Figure 3:**
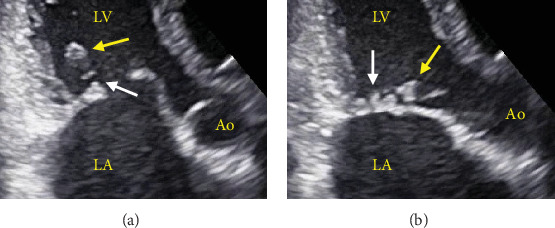
Two-dimensional TTE, zoomed apical 3-chamber view at late diastole (a) and mid-systole (b) demonstrates a mobile structure (*yellow arrows*) attached to the posterior mitral leaflet via a cord (*white arrows*). Ao, aorta; LA, left atrium; LV, left ventricle.

**Figure 4:**
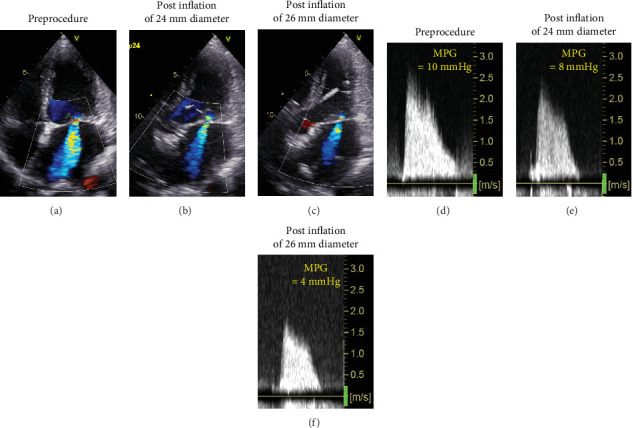
Monitoring of percutaneous mitral balloon valvuloplasty using TTE apical four-chamber view. Color Doppler flow and continuous-wave Doppler across the mitral valve before the procedure (a, d), after inflation with the 24-mm-diameter balloon, which was performed just after papillary muscle rupture occurred (b, e), and after the final inflation with the 26-mm-diameter balloon (c, f). These images demonstrate that there was no worsening of MR during the procedure, with an improvement in MS. MPG, mean pressure gradient; 3D-VCA, three-dimensional vena contracta area.

**Figure 5:**
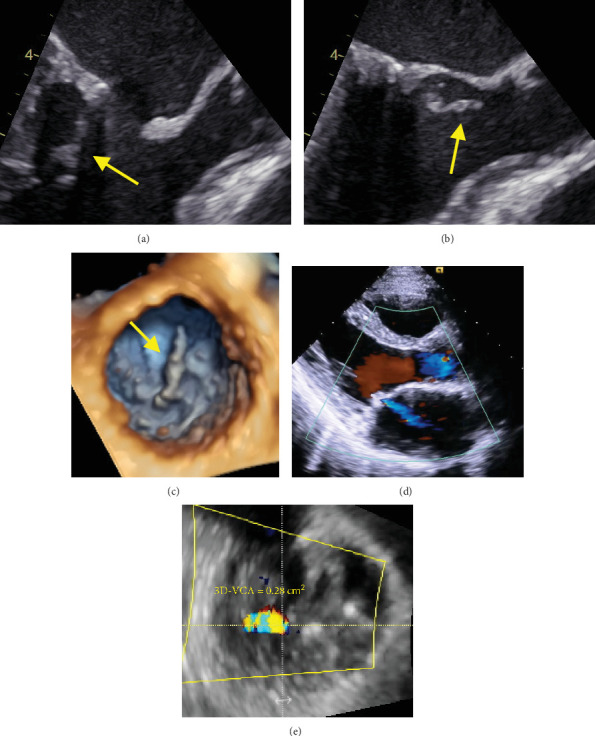
Echocardiographic images prior to patient discharge. Two-dimensional (2D) TEE long-axis zoomed view (120°) at late diastole (a) and mid-systole (b) and three-dimensional (3D) TEE en face view from the left ventricle (c) demonstrate a mobile structure attached to the posterior mitral leaflet (*yellow arrows*). 2D TTE color Doppler (d) and 3D TEE multiplanar reconstruction imaging (e) at mid-systole demonstrate central mild MR without leaflet prolapse.

The procedure was performed under local anesthesia with conscious sedation. The mean pressure gradient measured by TTE during the procedure was 10 mmHg at a heart rate of 90 beats/min with AT. After intracardiac echocardiography-guided and fluoroscopy-guided transseptal puncture, we attempted to insert the Inoue balloon catheter into the left ventricle under fluoroscopic guidance. Balloon mitral valvuloplasty was performed as previously described. First, only the distal half of the balloon was inflated in the left ventricle. Thereafter, the balloon was pulled until some resistance was felt. When we started further infusion quickly, we found a deep indentation of the balloon shortly followed by full extension of the balloon (Figure 2). Subsequently, TTE showed a mobile structure attached to the posterior mitral leaflet via a cord (Figure 3). We suspected a papillary muscle rupture. Fortunately, there was no worsening of MR. Therefore, we decided to proceed with the procedure to achieve a mean pressure gradient of less than 5 mmHg. Finally, the balloon was inflated to a size of 26 mm, and the mean pressure gradient decreased to 4 mmHg. Successful PTMC was confirmed by TTE, which showed no worsening of MR (Figure 4) and no complications, except for the papillary muscle rupture. The patient underwent cardioversion for persistent AF to restore sinus rhythm before leaving the operating room. The subsequent hospital course was uneventful, and she was discharged on the seventh postoperative day. On TTE and transesophageal echocardiography (TEE) prior to discharge, we observed a mobile mass with a cord-like structure connecting the posterior mitral leaflet, which we confirmed to be the papillary muscle with chordae tendinea (Figure 5). In addition, we did not observe any leaflet prolapse and worsening of MR (three-dimensional vena contracta area = 0.28 cm^2^) (Figure 5). Following the procedure, we decided to continue administering warfarin due to concerns about the recurrence of atrial arrhythmia caused by residual mild MS and MR. At the 6-month follow-up, the patient experienced an improvement in dyspnea on exertion with no worsening of MR or MS.

## 3. Discussion

Papillary muscle rupture is typically caused by acute myocardial infarction, and iatrogenic papillary muscle rupture is known as a rare but serious complication associated with transcatheter aortic valve replacement or implantation of temporary left ventricular assist devices [5–7]. In most cases, it leads to acute significant MR and subsequent acute life-threatening cardiogenic shock and pulmonary edema, requiring urgent surgical repair [8]. Papillary muscle rupture caused by the Inoue balloon during PTMC is relatively rare [8]. There have been several case reports of papillary muscle rupture following PTMC [4–6]. In contrast to our case with an uneventful follow-up without significant MR, patients in the previous reports all required surgical mitral valve intervention to manage acute severe MR, as in many other cases. Papillary muscles are connected to the mitral valve leaflets through the multiple chordae tendinea and functionally contract during systole, pulling the mitral valve leaflets against the left ventricular contractile force and preventing prolapse or flail of the valves [9]. Therefore, if the papillary muscle ruptures, the mitral leaflets lose their tensile strength and can flip back into the left atrium, resulting in prolapse or flail. However, in this case, the ruptured papillary muscle involved only the papillary muscle head stretching chordae tendinea to the only mitral posterior leaflet that was reduced in mobility due to the rheumatic changes; hence, papillary muscle rupture did not cause significant MR. Since we followed up our patient for only 6 months, long-term observation will be necessary to determine whether there is a worsening of MR.

In this case, the Inoue balloon suddenly inflated from an awkward shape with a slit. Upon straying into the complex chordae tendinea and papillary muscles when it was advanced into the left ventricle, the balloon got stuck in the complex after only the distal half of the balloon was inflated and was pulled back until some resistance was felt. We misinterpreted the image of poor dilatation to mean that the balloon was stuck in the commissural fusion of the mitral valve. If we had halted the inflation at a point where an abnormal shape was observed, with particular concern for chordae or papillary muscle rupture, the complication could have been avoided. Additionally, we retrospectively reviewed the intraoperative TTE image taken just before the inflation of the 24-mm Inoue balloon. This image revealed that the balloon was oriented toward the left aspect rather than directed toward the apex of the left ventricle, with much of the balloon encroaching upon the complex chordae tendinea (Figure S1). If we had paid more attention to this observation, we believe the rupture could have been prevented. Accurate monitoring using echocardiography during the procedure could help prevent complications.

## 4. Conclusions

We experienced a rare case where papillary muscle rupture occurred during PTMC, but there was no development of acute significant MR, and salvage surgery was not required. Careful observation using fluoroscopy and intraoperative echocardiography may prevent papillary muscle rupture.

## Data Availability

The data that support the findings of this study are available from the corresponding author upon reasonable request.

## Supporting Information

Supporting Information Additional supporting information can be found online in the Supporting Information section. Figure S1: The echocardiographic image just prior to the balloon inflation of 24 mm with TTE apical four-chamber view. It appeared that the balloon crossed the mitral valve with the tip facing the lateral side and strayed into the complex chordae tendinea (*yellow arrow*). LA, left atrium; LV, left ventricle.
