# Supplementary figures and images for: Papillary Muscle Rupture During Percutaneous Transvenous Mitral Commissurotomy: An Uncommon Scenario Exempt From Emergency Surgical Conversion
Source: Case Rep Cardiol. 2025 Aug 21;2025:5005780. doi: 10.1155/cric/5005780 (PMC12393946; doi:10.1155/cric/5005780)

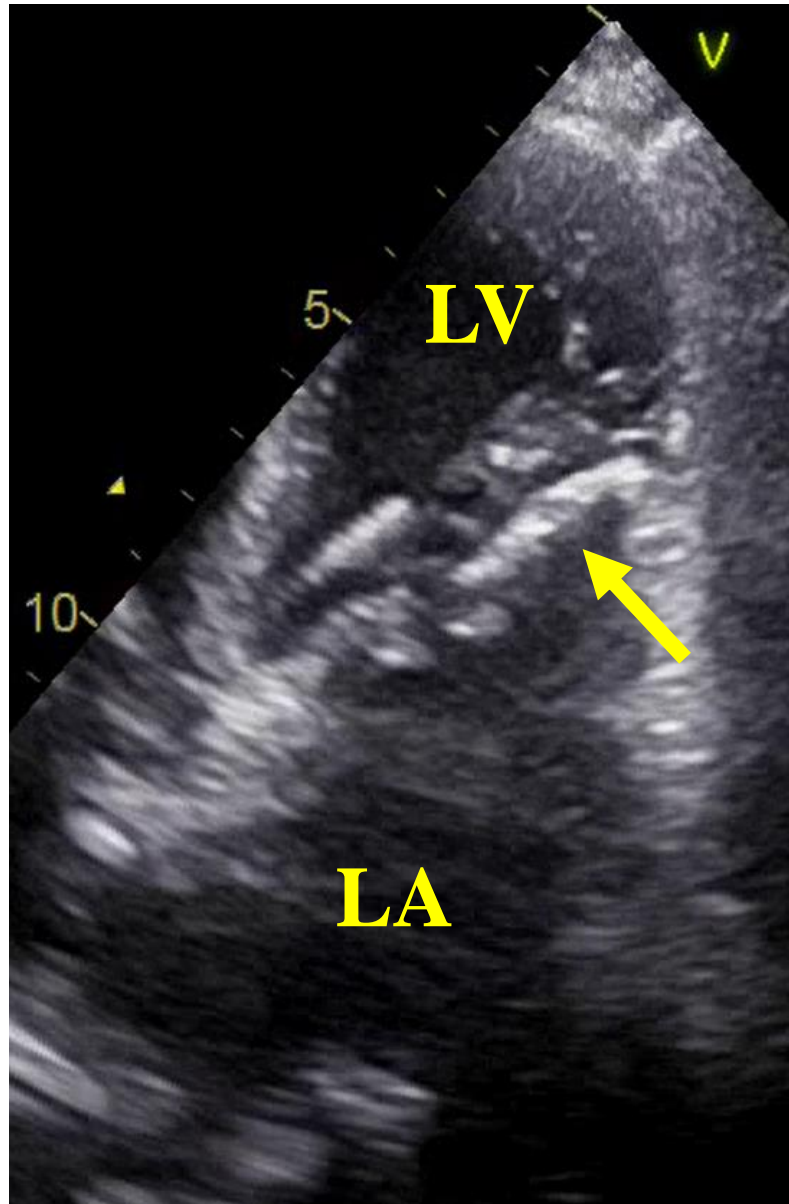

Figure S1

Supplement: Supporting Information — Additional supporting information can be found online in the Supporting Information section. Figure S1: The echocardiographic image just prior to the balloon inflation of 24 mm with TTE apical four-chamber view. It appeared that the balloon crossed the mitral valve with the tip facing the lateral side and strayed into the complex chordae tendinea (yellow arrow). LA, left atrium; LV, left ventricle. [file 5005780.f1.pdf]
